# Rapid and Ultrasensitive Short-Chain PFAS (GenX) Detection in Water via Surface-Enhanced Raman Spectroscopy with a Hierarchical Nanofibrous Substrate

**DOI:** 10.3390/nano15090655

**Published:** 2025-04-25

**Authors:** Ali K. Ismail, Shobha Mantripragada, Renzun Zhao, Sherine O. Obare, Lifeng Zhang

**Affiliations:** 1 Department of Nanoengineering, Joint School of Nanoscience and Nanoengineering, North Carolina A&T State University, Greensboro, NC 27401, USA; 2 Department of Civil, Architectural, and Environmental Engineering, College of Engineering, North Carolina A&T State University, Greensboro, NC 27411, USA; 3 Department of Nanoscience, Joint School of Nanoscience and Nanoengineering, University of North Carolina at Greensboro, Greensboro, NC 27401, USA

**Keywords:** short-chain PFASs, detection, surface-enhanced Raman scattering, electrospinning, nylon-6, Ag nanoparticles

## Abstract

GenX, the trade name of hexafluoropropylene oxide dimer acid (HFPO-DA) and its ammonium salt, is a short-chain PFAS that has emerged as a substitute for the legacy PFAS perfluorooctanoic acid (PFOA). However, GenX has turned out to be more toxic than people originally thought. In order to monitor and regulate water quality according to recently issued drinking water standards for GenX, rapid and ultrasensitive detection of GenX is urgently needed. For the first time, this study reports ultrasensitive (as low as 1 part per billion (ppb)) and fast detection (in minutes) of GenX in water via surface-enhanced Raman spectroscopy (SERS) using a hierarchical nanofibrous SERS substrate, which was prepared by assembling ~60 nm Ag nanoparticles on electrospun nylon-6 nanofibers through a “hot start” method. The findings in this research highlight the potential of the engineered hierarchical nanofibrous SERS substrate for enhanced detection of short-chain PFASs in water, contributing to the improvement of environmental monitoring and management strategies for PFASs.

## 1. Introduction

In response to the health hazards posed by per- and polyfluoroalkyl substances (PFASs) [1,2], regulatory measures have been introduced to limit their presence in the environment. These regulations have spurred increasing interest in the detection and monitoring of PFASs in water. Advanced techniques for PFAS detection, such as liquid chromatography-mass spectrometry, fluorescence spectroscopy, electrochemical detection, and Raman spectroscopy, have been explored by researchers in recent years [3,4,5,6]. Raman spectroscopy has demonstrated the capability to distinctly characterize molecules based on their unique spectral fingerprints, and it shows promise for effectively monitoring and managing water pollutants, on account of its speediness [7]. However, the normally weak Raman signal of low-concentration molecules in water necessitates the technique of surface-enhanced Raman spectroscopy (SERS). SERS exploits the amplification of Raman signals of molecules by up to 10^10^ times, with the possibility of single-molecule detection [6], typically by using silver (Ag) or gold (Au) nanoparticle clusters, based on the enhanced electromagnetic field in the vicinity of adjacent nanoparticles (termed as hotspots) due to their localized surface plasma resonance. The key advantage of SERS lies in its ability to precisely identify target molecules in a speedy, non-destructive, and cost-effective way [8].

However, the use of SERS to detect PFASs in water is still in its infancy. To date, there are only a handful of reports for the most studied PFASs, including perfluorooctanoic acid (PFOA) [3,4,5,6,9], perfluorooctane sulfonic acid (PFOS) [5,6], and a few others, such as perfluorohexanoic acid (PFHxA) and potassium perfluorobutanesulfonate (PPFBS) [3]. Obstacles include the ultra-low concentration of PFAS molecules in water and, correspondingly, the challenge of SERS substrate development for highly sensitive PFAS detection.

GenX, the trade name of hexafluoropropylene oxide dimer acid (HFPO-DA) and its ammonium salt, is a short-chain (six carbon) PFAS that has been used as a replacement for PFOA, with the expectation that it would reduce the health concerns surrounding PFOA; however, it turned out to be more toxic than people originally thought [10,11,12]. The U.S. EPA issued the first ever stringent national drinking water standard for GenX in April 2024. Developing an effective SERS substrate for sensitive, reliable, and rapid detection of GenX in water is an urgent need in order to monitor its presence in different water bodies.

Ag nanoparticles (AgNPs) have been widely used for SERS purposes, due to their high SERS enhancement capability, cost-effectiveness, and ease of synthesis [13]. According to our previous research [14], growing AgNPs on appropriate electrospun nanofibers could be an advantageous way to realize an effective SERS substrate for GenX detection in water. In this study, we sought to fill the gap in the literature and contribute to the development of a sensitive, cost-effective, and flexible monitoring platform for the emerging contaminant of GenX, by identifying specific Raman peaks that are associated with GenX and picking an appropriate material for electrospun nanofibers, followed by managing the size, shape, and distribution of AgNPs on electrospun nanofibers for SERS hotspot construction. It is noteworthy that this is the first time that detection of ultra-low concentration GenX in water has been achieved with a hierarchical electrospun nanofibrous SERS substrate. The flexible SERS substrate developed in this research is suitable for scalable integration with wipes or swabs to collect water samples for GenX sensing, and thus has great potential for monitoring GenX in different water bodies through mobile and field testing.

## 2. Materials and Methods

### 2.1. Materials

Acetic acid (CH_3_COOH, 99.7%), formic acid (HCOOH, 95%), N,N-dimethylformamide (DMF, 99%), sodium citrate (Na_3_C_6_H_5_O_7_), silver nitrate (AgNO_3_), and hydrochloric acid (HCl, 1 M) were purchased from Sigma Aldrich (Louis, MO, USA). Nylon-6 was purchased from Fisher Chemical (Durham, NC, USA). GenX was purchased from Manchester Organics (Runcorn, Cheshire, UK).

### 2.2. Preparation of Electrospun Nylon Nanofibers

The electrospinning solution was prepared by dissolving nylon-6 pellets in a formic acid/acetic acid solvent. The final clear and homogeneous solution, containing 14 wt.% nylon, 64 wt.% formic acid, and 22 wt.% acetic acid, was loaded to a Spinbox electrospinning unit (Nanoscience Instruments, Phoenix, AZ, USA) and electrospun for 4 h, at a flow rate of 0.2 mL/h, under an applied voltage of 23 kV. A 23-gauge blunt-end needle was used, with a needle-to-collector distance of 12 inches. After electrospinning, the nylon nanofibrous mat deposited on the aluminum foil that wrapped the rotating collector was carefully removed and allowed to air-dry for three days.

### 2.3. Assembly of Silver Nanoparticles on Nylon Nanofibers

First, 22.5 mg AgNO_3_ was weighed and dissolved in 100 mL of DI water. The solution was magnetically stirred with a stirring bar at 800 rpm on a hotplate and heated to boiling state. A 2.5 mL volume of 2 wt.% sodium citrate solution was then added to the AgNO_3_ solution. Next, the heat was removed, and the reaction was allowed to continue until the solution turned to a dark brown color with a slight silver tinge. Subsequently, the solution was cooled down to room temperature, and its pH was adjusted to pH = 5 using HCl. After that, 25 mL of the acquired Ag precursor solution was poured into a beaker, and a 1 in. × 1 in. square piece of electrospun nylon nanofibrous mat was immersed in the solution. The pH of the solution was further reduced to pH = 4 and monitored with a pH meter. The beaker was covered with wax paper afterwards, and kept in a dark environment for 24 h to let AgNPs assemble on the nanofiber surface. The acquired nylon-6/AgNPs nanofibrous SERS substrate (termed as ES nylon-6/AgNPs) was thoroughly rinsed with DI water and left to dry at room temperature.

### 2.4. Characterization

The morphology of the ES nylon-6/AgNPs was examined with a JEOL (Peabody, MA, USA) JSM-IT800 Schottky field-emission scanning electron microscope (FESEM), equipped with an energy-dispersive X-ray spectrometer (EDS), at an accelerating voltage of 15 kV and a working distance of 10 mm. The SEM samples were mounted onto respective SEM pin stubs using double-sided carbon tape and sputter-coated with a 6 nm Au/Pd layer to enhance the imaging quality. AgNP size distribution analysis was performed using ImageJ software (version 1.8.0) by measuring 400 random particles in SEM images, and the resulting data were used to generate a histogram through OriginPro software (version 2023b). Raman spectroscopy was carried out by using a Horiba (Irvine, CA, USA) XploRA Raman Confocal Microscope equipped with a 535 nm, 25 mW monochromatic green laser, which can achieve up to 1 µm XY resolution. SERS testing of individual samples was operated at 1% laser power, with five accumulations of 5 s each, and run multiple times to ensure accuracy and repeatability. To assess the detection limit of GenX, 25 µL of GenX solutions at different concentrations were applied to the SERS substrate, and Raman scans were performed with progressively lower concentrations until no signal was detected. Standard GenX solutions with concentrations ranging from 1000 ppm to 10 ppt were prepared by dissolving 1.0 g GenX in 100 mL water, followed by serial dilutions using a micropipette. The Raman spectra were presented in figures, with the intensity normalized/adjusted for appropriate comparison purposes.

## 3. Results and Discussion

### 3.1. Determining the Unique Fingerprint Raman Signal of GenX Molecules

**Figure 1:**
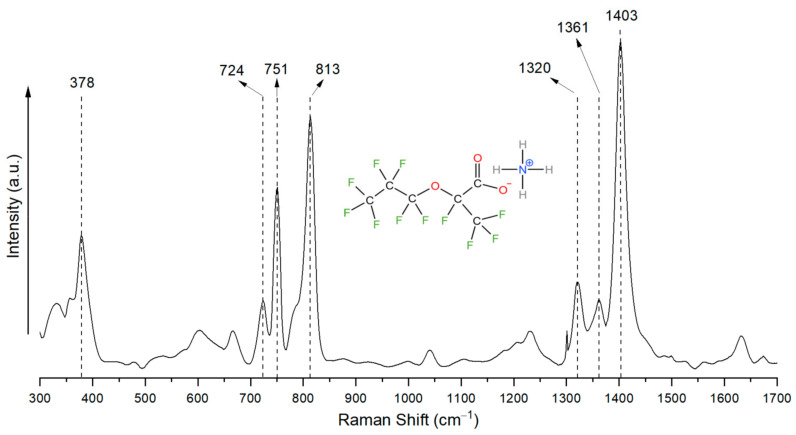
Raman spectrum of solid GenX powder.

Even though GenX has been the focus of several research papers, there is currently a lack of published work addressing its Raman spectrum. Therefore, understanding the Raman spectral signatures of GenX in its pure solid state is necessary to ensure accurate identification of GenX in water. The Raman spectrum of GenX powder is shown in Figure 1.

In the Raman spectrum of GenX, the 378 cm^−1^ peak could be assigned to –CF_2_–. As reported, –CF_2_– typically exhibits deformation vibrations around this frequency [15]. The 724 cm^−1^ peak could be similarly linked to –CF_2_– bonding, though some sources have debated whether it could stem from –CF_3_ or even C–C bonds [5,6,15]. Given the strength of this peak relative to that of others in the spectrum, it is more likely a combination of the abovementioned structures. This inference aligns with other studies of short-chain PFAS molecules like perfluorobutanoic acid (PFBA), which has exhibited similar activity in this Raman shift region [16]. The peak at 751 cm^−1^ could be caused by a combination of –CF_2_– and –CF_3_ bonding [5,6,16]. The 813 cm^−1^ peak could also be assigned to –CF_3_ [6]. A GenX molecule contains two –CF_3_ groups and two –CF_2_– groups, and thus resulted in strong peaks at 751 cm^−1^ and 813 cm^−1^. The 813 cm^−1^ peak is notable because, although other PFAS molecules also have peaks around 805 cm^−1^, the nearby C–O–C bond on the skeletal chain of GenX molecule may have led to the shift to 813 cm^−1^ [5,6,16,17]. The overall signal contributed by –CF_3_ and –CF_2_– bonds with the nearby C–O–C bond makes the two peaks at 751 cm^−1^ and 813 cm^−1^ critical markers for GenX molecules. In the higher-wavenumber region, the 1320 cm^−1^ peak could be assigned to –CF_3_ and –CF_2_– groups, and the 1361 cm^−1^ peak could be attributed to a combination of –CF< and –(C=O)–O (carboxylate) groups [6,8,18]. The significant peak at 1403 cm^−1^ could be attributed to the combination of –CF_3_, as well as NH_4_^+^, originating from the ammonium salt of the GenX molecule [6,18]. All of these peak assignments established a comprehensive Raman fingerprint for the GenX molecule, distinguishing it from other PFAS molecules such as PFOS, PFOA, and PFBA [5,6], particularly by using the unique combination of three strongest peaks of –CF_3_, –CF_2_–, C–O–C, and –COO^-^ at wavenumbers of 751 cm^−1^, 813 cm^−1^, and 1403 cm^−1^.

**Figure 2:**
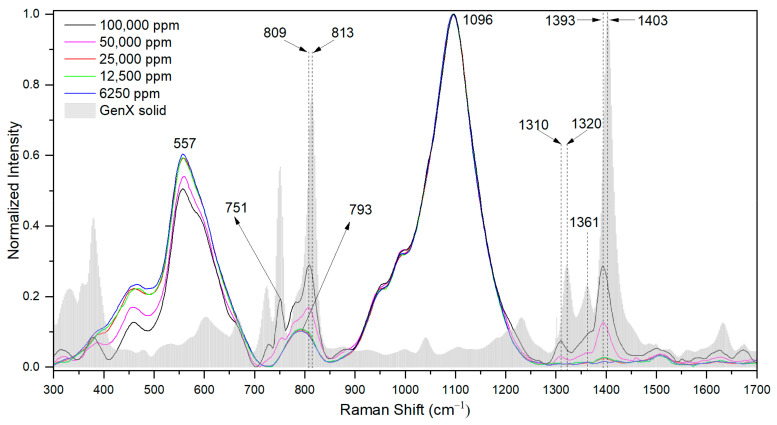
Raman spectra of GenX water at concentrations of 100,000 ppm, 50,000 ppm, 25,000 ppm, 12,500 ppm, and 6250 ppm. All the spectra were normalized by the 1096 cm^−1^ peak. The Raman spectrum of solid GenX powder is shown as a reference.

To examine the Raman signal from GenX in water, Raman scans were carried out by using a drop of GenX water with different concentrations, from 100,000 ppm down to 6250 ppm, on a piece of glass slide (Figure 2).

In aqueous solution, some Raman peaks of GenX molecules exhibited slight shifts compared to those from solid state form. The notable shifts included 813 cm^−1^ → 809 cm^−1^, 1320 cm^−1^ → 1310 cm^−1^, 1361 cm^−1^ → not identifiable (n.i.), and 1403 cm^−1^ → 1393 cm^−1^. All these peak shifts indicate molecular conformation changes of GenX, likely due to the dissociation of NH_4_^+^ from the GenX molecule in water, as well as the interaction between GenX molecules and water molecules [19]. Overall, seven major Raman shift peaks of GenX molecules and their respective bond assignments were cross-referenced with the literature, and are shown in Table 1.

It is noteworthy that the glass slide exhibited strong Raman signals centered at 557 cm^−1^, 793 cm^−1^, and 1096 cm^−1^, which overlapped with the Raman signals of GenX. In particular, the 793 cm^−1^ Raman shift peak could interfere with the characteristic GenX peaks at 751 cm^−1^ and 809 cm^−1^. By using the GenX characteristic Raman shift peak at 1393 cm^−1^, we could confidently detect GenX in water by using a drop of GenX water on a glass slide at a concentration of 50,000 ppm. Below this concentration, reliable Raman peaks diminished, underscoring the necessity of SERS for detecting GenX at lower concentrations.

### 3.2. Highly Sensitive GenX Detection in Water Using SERS

**Figure 3:**
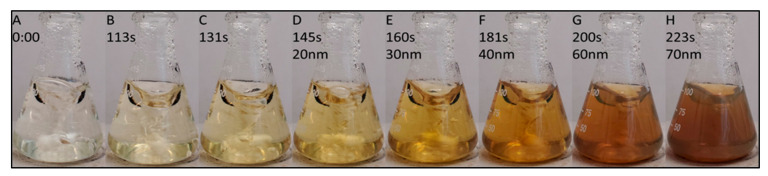
Relationship between reaction time (coloration) and AgNP size using sodium citrate-driven redox reaction.

**Figure 4:**
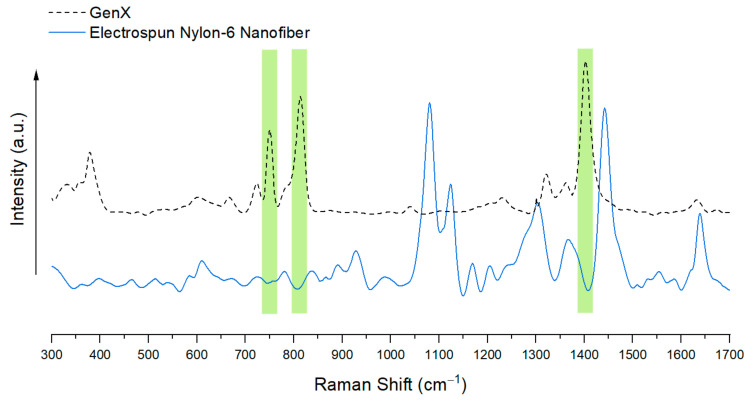
Comparison of Raman spectra of GenX solid and electrospun nylon-6 nanofibers.

Although gold nanoparticles have also been used as a SERS substrate, AgNPs offer a more cost-effective option. AgNPs have demonstrated a SERS enhancement factor of up to 10^10^, granting them the capability of single molecule detection [20]. In this study, a redox reaction of AgNO_3_ with sodium citrate in DI water was employed, which allowed for tunable nanoparticle sizes that could be easily characterized by the color of the reaction solution, as shown in Figure 3. By placing a piece of nylon-6 electrospun nanofibrous mat into the AgNP-growing solution, the desired AgNP assembly on nanofibers, with tunable nanoparticle sizes, was realized. Herein, nylon-6 was selected as the electrospun nanofiber material, because of the transparency of GenX Raman signals at 751 cm^−1^, 813 cm^−1^, and 1403 cm^−1^ when comparing the Raman signals of GenX and those of electrospun nylon-6 nanofibers (Figure 4).

**Figure 5:**
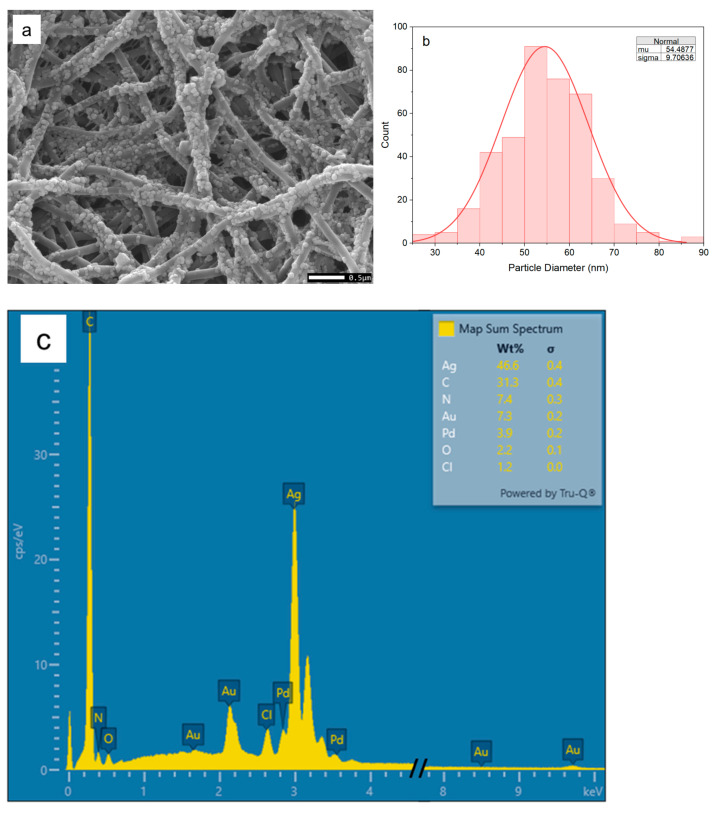
(**a**) SEM image, (**b**) AgNP size distribution, and (**c**) EDS spectrum of ES nylon-6/AgNPs SERS substrate.

Based on numerous trials, a hierarchical SERS substrate with outstanding SERS activity, i.e., electrospun nylon-6 fibers with surface-attached AgNPs (ES nylon-6/AgNPs) (Figure 5a), was obtained through a “hot start” method, as described in the Experimental Section. The average size of the synthesized AgNPs was determined to be 54.5 ± 9.7 nm (Figure 5b). The Ag element was confirmed by EDS analysis (Figure 5c). The presence of Au and Pd was due to the sputter coating of Au-Pd for good SEM imaging.

**Figure 6:**
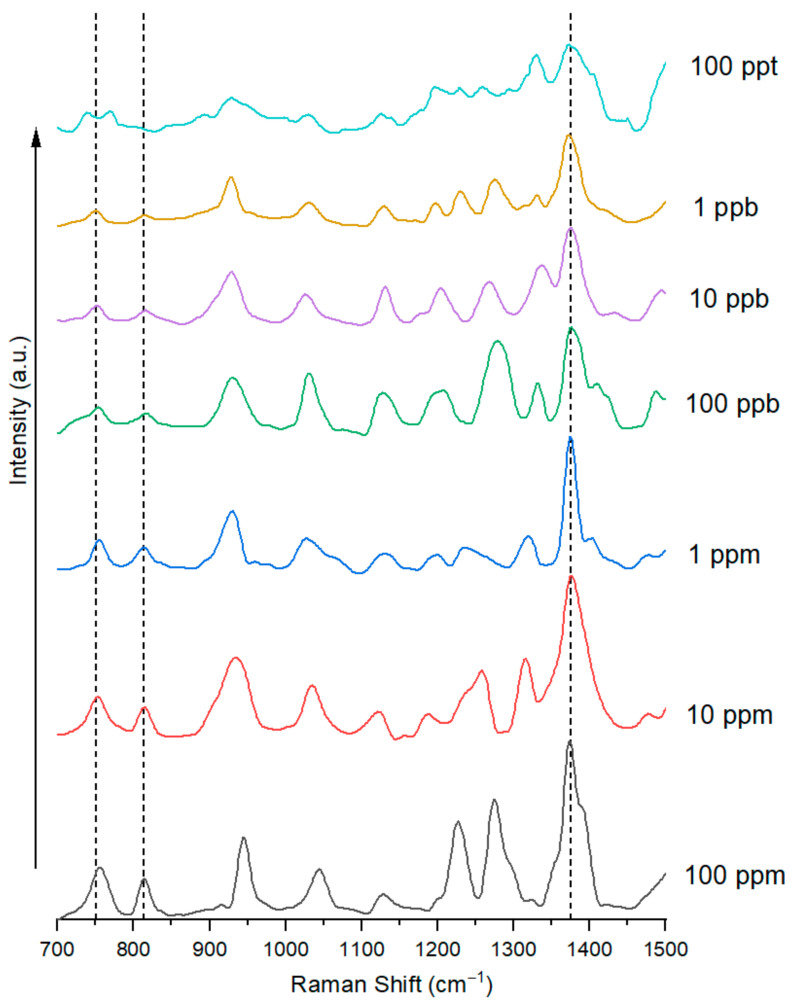
Raman spectra (700–1500 cm^−1^) of GenX water with GenX concentrations ranging from 10 ppm to 100 ppt (2.88 × 10^−4^ mol/L to 2.88 × 10^−10^ mol/L) on the hierarchical ES nylon-6/AgNPs SERS substrate.

In the range of GenX concentration from 100 ppm (mg/L) to 100 ppt (ng/L), i.e., 2.88 × 10^−4^ mol/L to 2.88 × 10^−10^ mol/L, two GenX concentration-dependent Raman peaks were identified in the Raman spectra of GenX water on the hierarchical ES nylon-6/AgNP nanofibrous substrate: 749–755 cm^−1^ and 813–816 cm^−1^ (Figure 6). These two peaks are consistent with the previously identified GenX characteristic Raman shift peaks at 751 cm^−1^ and 809–813 cm^−1^. It is noteworthy that the strong 1393 cm^−1^ Raman shift peak of GenX disappeared with the ES nylon-6/AgNPs substrate. A nearby peak at ~1373 cm^−1^ also appeared to be GenX concentration-dependent, but it overlapped with nylon-6’s 1367 cm^−1^ Raman peak (Figure 4), and should not be considered as a very reliable quantitative indicator of GenX presence.

**Figure 7:**
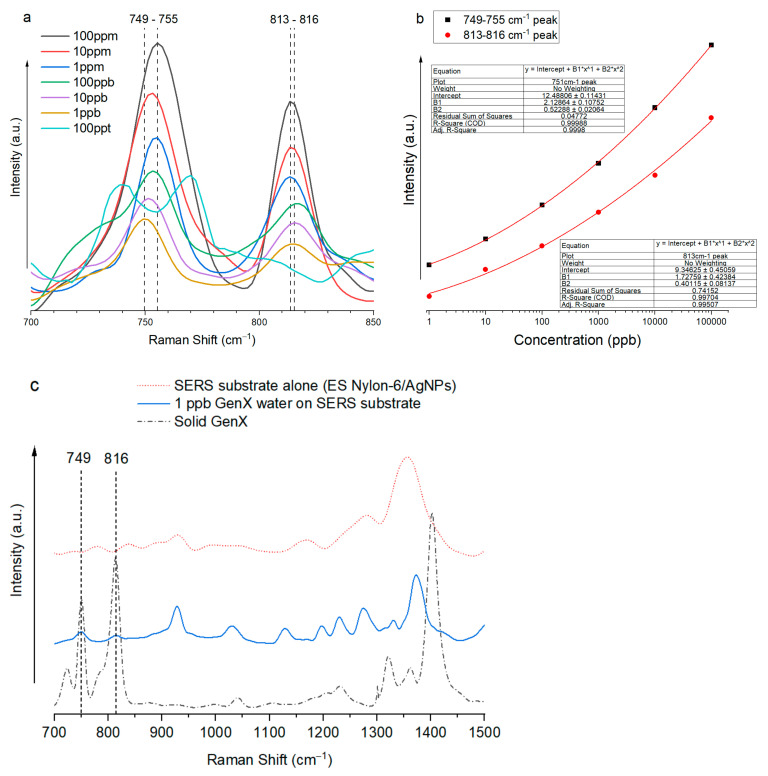
(**a**) Raman spectra of GenX water with GenX concentrations ranging from 10 ppm to 100 ppt (2.88 × 10^−4^ mol/L to 2.88 × 10^−10^ mol/L) on the ES nylon-6/AgNPs SERS substrate, with the focus on Raman shift peaks at 749–755 cm^−1^ and 813–816 cm^−1^; (**b**) Polynomial fitting between the enhanced GenX Raman signals at these two Raman shifts and the log (GenX concentration); (**c**) A comparison of the Raman spectra (700–1500 cm^−1^) of solid GenX, 1 ppb GenX water on the SERS substrate, and the SERS substrate alone (ES Nylon-6/AgNPs).

The GenX Raman shift peaks at ~751 cm^−1^ and ~813 cm^−1^ were discernable even at 1 ppb concentration (Figure 7a). The relationship between the enhanced GenX Raman signals at these two positions and the log10 of GenX concentration followed very well the second-degree polynomial of log (GenX concentration), with R^2^ = 0.99988 for the ~751 cm^−1^ peak and R^2^ = 0.99704 for the ~813 cm^−1^ peak (Figure 7b).

To check if the SERS substrate alone had any influence on GenX detection, a direct Raman signal comparison of solid GenX, 1 ppb GenX water on the ES nylon-6/AgNP SERS substrate, and the ES nylon-6/AgNP SERS substrate alone without GenX, was conducted, and the results are shown in Figure 7c. The Raman signal from the SERS substrate acted as a control. It is clear from Figure 7c that there was no appreciable influence from the ES nylon-6/AgNP substrate on GenX detection using the characteristic Raman shift peaks of GenX at ~751 cm^−1^ and ~813 cm^−1^.

Ultimately, the GenX limit of detection (LOD) using the prepared ES nylon-6/AgNP SERS substrate was convincingly determined to be 1 ppb, based on consistent presence of key GenX Raman shift peaks at ~751 cm^−1^ and ~813 cm^−1^ through multiple Raman runs, which could be attributed to the –CF_3_, –CF_2_–, and C–O–C structures in GenX molecule. The signal enhancement factor (EF) was calculated using the lowest detectable GenX concentration and its corresponding Raman signal intensity with and without the SERS substrate. The calculated result for the EF based on the 751 cm^−1^ peak was 1.3 × 10^7^.

$$EF=\frac{I_{SERS}}{I_{Raman}}\times \frac{C_{Raman}}{C_{SERS}}=\frac{12.433}{49.619}\times \frac{50,000\;ppm}{0.001\;ppm}=1.3\times 10^{7}$$

Interestingly, we did not observe significant Raman signal amplification with other sizes of AgNPs on electrospun nylon-6 nanofibers, including those with average sizes < 50 nm or >80 nm. These sizes of Ag nanoparticles yielded negligible or no GenX signals. It is well known that the assembly of AgNPs can significantly influence SERS sensitivity [14]. The SERS active substrate we prepared in this study contained AgNPs with an average size ~60 nm, which aligns with a few previous reports of 60 nm AgNPs demonstrating excellent SERS activity [20,21,22]. The assembly of 60 nm AgNPs on electrospun nylon-6 nanofibers may be able to construct rich hotspots, and thus benefit the detection of GenX at ultra-low concentrations. In this research, the AgNPs were synthesized with citrate. The presence of citrate on the AgNP surface could have been involved in hydrogen bonding between the carboxylic acid groups of citrate molecules and the amide groups of nylon-6, and thus prevented AgNPs from agglomeration [23,24], subsequently facilitating SERS hotspot formation.

## 4. Conclusions

In this study, ultrasensitive detection of GenX (the ammonium salt of hexafluoropropylene oxide dimer acid (HFPO-DA), a short-chain PFAS) was achieved via surface-enhanced Raman spectroscopy (SERS) by identifying the Raman fingerprint for GenX molecules and assembling an appropriate hierarchical nanofibrous substrate. Specifically, the SERS substrate of ES nylon-6/AgNPs, i.e., electrospun nylon-6 nanofibers with surface-attached silver nanoparticles (average size: 54.5 ± 9.7 nm), demonstrated excellent capability for GenX detection in ultra-low-concentration aqueous solution with a limit of detection (LOD) at 1 part per billion (ppb) by using the characteristic Raman shift peaks of GenX molecules at ~751 cm^−1^ and ~813 cm^−1^, which could be attributed to –CF_3_, –CF_2_–, and C–O–C structures in GenX molecules. The relationship between the enhanced GenX Raman signals at these two Raman shifts and the log base 10 of GenX concentration followed very well the second-degree polynomial, with R^2^ = 0.99988 for the ~751 cm^−1^ Raman shift and R^2^ = 0.99704 for the ~813 cm^−1^ Raman shift. The SERS enhancement factor (EF) of the ES nylon-6/AgNPs substrate reached 1.3 × 10^7^ based on the ~751 cm^−1^ Raman shift of GenX. This research demonstrates great promise for the use of SERS to address the critical environmental and health concerns associated with short-chain PFAS contamination, opening up avenues for the development of sensitive, reliable, portable, and rapid PFAS detection systems to safeguard our water resources and communities.

## Figures and Tables

**Table 1:** Raman shift peak assignment of GenX molecule.

Raman Shift for Solid State (cm^−1^)	Raman Shift When Dissolved in Water (cm^−1^)	Chemical Bond Assignment	Reference
378	378	–CF_2_–	[15]
724	728	C–C, –CF_2_–, –CF_3_	[5,6,15]
751	751	–CF_3_, –CF_2_–	[6,16,17]
813	809	–CF_2_–,–CF_3_, –C–O–C–	[5,6,16,17,18]
1320	1310	–CF_2_–, –CF_3_	[5,18]
1361	n.i.	–CF<, –COO^−^	[6,18]
1403	1393	–CF_3_, NH_4_^+^	[6,18]

## Data Availability

The original contributions presented in this study are included in the article. Further inquiries can be directed to the corresponding author.
